# Tailoring high-refractive-index nanocomposites for manufacturing of ultraviolet metasurfaces

**DOI:** 10.1038/s41378-024-00681-w

**Published:** 2024-04-22

**Authors:** Hyunjung Kang, Dongkyo Oh, Nara Jeon, Joohoon Kim, Hongyoon Kim, Trevon Badloe, Junsuk Rho

**Affiliations:** 1 Department of Mechanical Engineering, Pohang University of Science and Technology (POSTECH), Pohang, Republic of Korea; 2 Graduate School of Artificial Intelligence, Pohang University of Science and Technology (POSTECH), Pohang, Republic of Korea; 3 Department of Chemical Engineering, Pohang University of Science and Technology (POSTECH), Pohang, Republic of Korea; 4 Department of Electrical Engineering, Pohang University of Science and Technology (POSTECH), Pohang, Republic of Korea; 5 POSCO-POSTECH-RIST Convergence Research Center for Flat Optics and Metaphotonics, Pohang, Republic of Korea; 6 National Institute of Nanomaterials Technology (NINT), Pohang, Republic of Korea

**Keywords:** Nanophotonics and plasmonics, Nanoparticles, Structural properties, Micro-optics

## Abstract

Nanoimprint lithography (NIL) has been utilized to address the manufacturing challenges of high cost and low throughput for optical metasurfaces. To overcome the limitations inherent in conventional imprint resins characterized by a low refractive index (*n*), high-*n* nanocomposites have been introduced to directly serve as meta-atoms. However, comprehensive research on these nanocomposites is notably lacking. In this study, we focus on the composition of high-*n* zirconium dioxide (ZrO_2_) nanoparticle (NP) concentration and solvents used to produce ultraviolet (UV) metaholograms and quantify the transfer fidelity by the measured conversion efficiency. The utilization of 80 wt% ZrO_2_ NPs in MIBK, MEK, and acetone results in conversion efficiencies of 62.3%, 51.4%, and 61.5%, respectively, at a wavelength of 325 nm. The analysis of the solvent composition and NP concentration can further enhance the manufacturing capabilities of high-*n* nanocomposites in NIL, enabling potential practical use of optical metasurfaces.

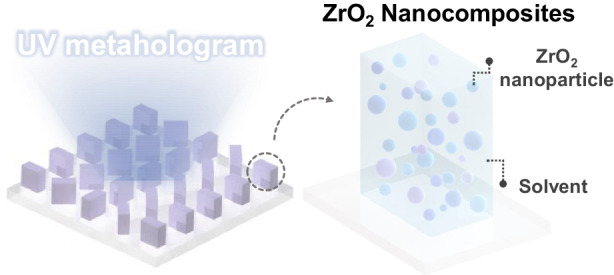

## Introduction

Metasurfaces are composed of subwavelength structures in a two-dimensional arrangement and have electromagnetic (EM) properties that can be engineered for applications such as holography^1–8^, lenses^9–13^, biosensors^14–17^, and structural color printing^18–23^. Ultraviolet (UV) metasurfaces exhibit distinct characteristics that enable control of UV light in various applications, including imaging^24–26^, display^27,28^, and biosensing^29,30^. However, as UV metasurfaces must have structures that are significantly smaller than the wavelength of UV radiation, costly nanopatterning techniques are required to achieve nanoscale resolution and high aspect ratios, with additional challenges related to the limited selection of materials with high transparency in the UV region. Metasurfaces are usually fabricated using well-established methods such as atomic layer deposition and electron-beam lithography (EBL), which achieve high resolution; however, the patterning area is small, the operational costs are very high, and additional complex processing steps may be required, making these approaches unsuitable for mass production. Nanoimprint lithography (NIL) has emerged as a solution to overcome these limitations for practical applications^31–35^. Nevertheless, the inherently low refractive index (*n*) of the conventional imprint resins (≈1.5) commonly employed in NIL results in a low conversion efficiency of the metasurfaces^36^.

To address this challenge, a one-step NIL fabrication method for metasurfaces utilizing high-*n* nanocomposites of dielectric nanoparticles (NPs) to enhance the effective *n* of the printed structures while replicating mold patterns as effectively as conventional resins has been introduced^37–43^. The choice of NP depends on the target wavelength range; for instance, titanium dioxide (TiO_2_)^44^ and silicon (Si)^45^ NPs have been utilized for the visible and infrared (IR) regions, respectively. Notably, a straightforward printing platform employing zirconium dioxide (ZrO_2_) to realize high-efficiency metaholograms that function in the near-to-deep UV range has recently been demonstrated^46^. As the high-*n* nanocomposite is directly employed as meta-atoms, research on the composition (i.e., NP concentration, solvent, etc.) needed to achieve high-fidelity metasurfaces is significant but lacking.

Understanding the influence of the diverse constituents in nanocomposites on the transfer fidelity is crucial for establishing a robust and reliable metasurface fabrication process. Each type of NP exhibits variations in *n*, the adhesion strength, and the dispersibility in solvents, necessitating the development of distinct fabrication conditions^47,48^. For example, the maximum NP concentration is limited due to particle agglomeration, and the interactions between the solvent and mold, such as swelling and surface roughness, must be considered. In this study, we investigate the impact of the NP concentration and solvent selection on the transfer fidelity, which is evaluated through the conversion efficiency of metaholograms replicated using ZrO_2_ nanocomposites. We compare the metasurfaces printed with ZrO_2_ NP concentrations ranging from 20 to 90 wt% to establish the optimal NP concentration to maximize the effective *n* (≈ 1.8 at λ = 325 nm) with high transfer fidelity. Subsequently, while maintaining the NP concentration at the determined value, we explore different solvents, including methyl isobutyl ketone (MIBK), methyl ethyl ketone (MEK), acetone, toluene, and n-hexane. As a demonstration of the tailored ZrO_2_ nanocomposites, the conversion efficiencies of metaholograms operating at 325 nm are experimentally measured to quantify the transfer fidelity. These findings hold promise for refining the fabrication process and broadening the potential applications of nanocomposites in NIL.

## Results

**Fig. 1:**
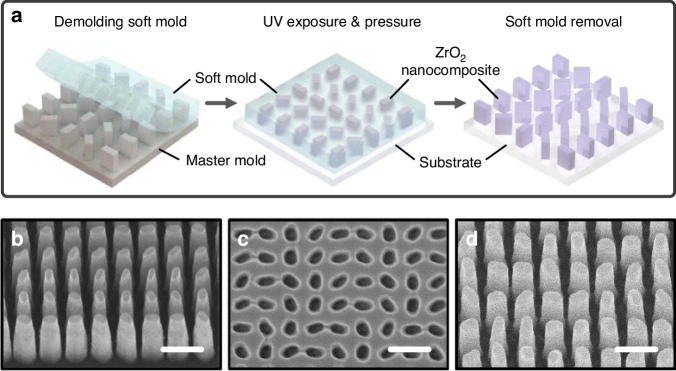
**a** Schematic configuration of nanoimprint lithography (NIL) using zirconium dioxide (ZrO_2_) nanocomposites. Scanning electron microscope (SEM) images of **b** the master mold, **c** the soft mold, and **d** replicated ultraviolet (UV) metaholograms. All scale bars: 300 nm

**Fig. 2:**
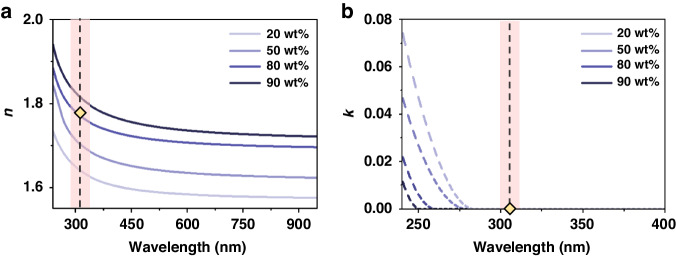
**a** Refractive index (*n*) and **b** extinction coefficient (*k*) according to the ZrO_2_ nanoparticle (NP) concentration

A schematic of the NIL process employed to fabricate UV metaholograms using ZrO_2_ nanocomposites is depicted in Fig. 1a (details in the *Materials and Methods*). To generate metaholograms operating at λ = 325 nm, first, a master mold is fabricated using the standard EBL process (Fig. 1b). This master mold is then covered with a hard-polydimethylsiloxane (h-PDMS)/PDMS bilayer and cured to form a soft mold (Fig. 1c). A uniform ZrO_2_ nanocomposite film is then coated on the soft mold, and then, pressure and UV exposure are applied. The soft mold is detached to leave the UV metahologram on the substrate (Fig. 1d). Nanocomposite films with different ZrO_2_ NP concentrations are uniformly coated onto a glass substrate, and the *n* and extinction coefficient (*k*) are measured using ellipsometry (Figs. S1, S2). The results are shown in Fig. 2a, b. As expected, higher ZrO_2_ NP concentrations yield higher values of *n*, while in the deep-UV region (~280 nm), the measured *k* is suppressed for higher ZrO_2_ NP concentrations and is consistently zero at 325 nm.

**Fig. 3:**
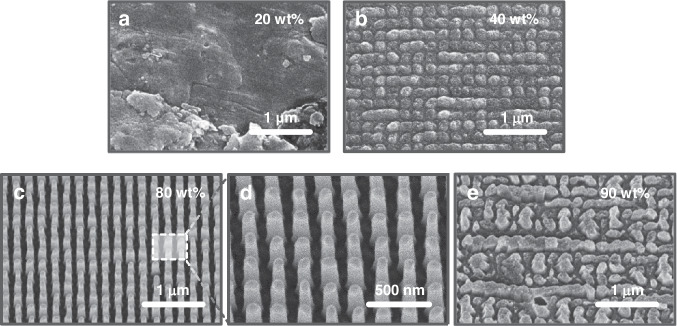
UV metaholograms replicated using the **(a)** 20 wt%, **(b)** 50 wt%, **(c, d)** 80 wt%, and **(e)** 90 wt% ZrO_2_ nanocomposites

Optimizing the NP concentration is essential for closely mimicking the shape of the master mold. At 20 and 50 wt%, there are not enough NPs to adequately support the structures during imprinting, leading to low transfer fidelity (Fig. 3a, b). At 80 wt%, the optimal conditions are met, as enough NPs are present to create the desired structure without any particle agglomeration (Fig. 3c, d). Conversely, at 90 wt%, the NP concentration is excessive, and particle agglomeration occurs within the nanocomposite, leading to clusters larger than the cavity size of the soft mold. This disrupts the formation of the intended structures, yielding indistinct or inaccurate patterns (Fig. 3e). These results highlight the critical parameter of the NP concentration in the nanocomposite for achieving high transfer fidelity and structural stability.

**Fig. 4:**
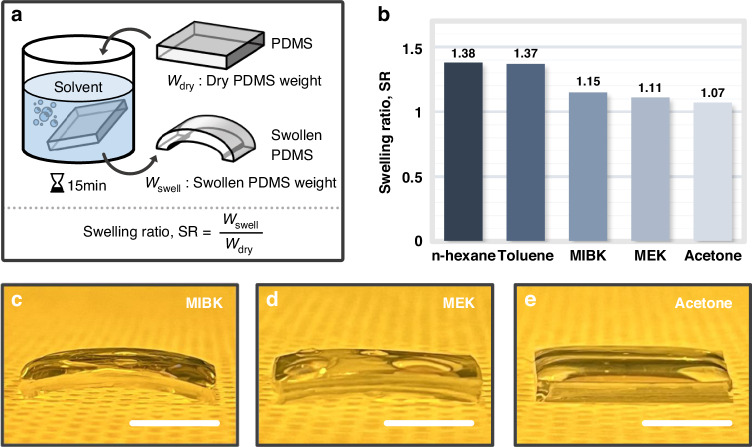
**a** Schematic configuration of the experimental procedure for measuring the swelling ratio of polydimethylsiloxane (PDMS). **b** Calculated swelling ratios for each solvent. Photographs of swollen PDMS after soaking in **c** methyl isobutyl ketone (MIBK), **d** methyl ethyl ketone (MEK), and **e** acetone. All scale bars: 1 cm

During the NIL process, swelling occurs when the nanocomposite film is coated on the soft mold, thus degrading the quality of the resin layer, as it distorts the form in the soft mold^49,50^. Although the influence of swelling on NIL is widely recognized, more research and quantitative evaluation are needed. MIBK has been used as a solvent for nanocomposites due to its effective resin dissolution properties^44–46^. However, swelling of the soft mold is apparent during the replication process, which is attributed to interactions between the solvent and the PDMS, leading to fabrication imperfections that can negatively affect the functionality of the replicated nanostructures. To explore potential solvents for high-*n* nanocomposites, n-hexane, toluene, MEK, and acetone are assessed (details in the *Materials and Methods*) with regard to PDMS swelling, which is defined as the ratio between the weight of the swollen PDMS (*W*_*swell*_) and its initial dry weight (*W*_*dry*_) (Fig. 4a). The measured weights are provided in Table S1. MEK and acetone exhibit swelling ratios of 1.11 and 1.07, respectively, which are both lower than the corresponding value of 1.15 for MIBK (Fig. 4b). These values are visualized in images of the apparent swelling of the PDMS soaked in MIBK, MEK, and acetone (Fig. 4c–e). While maintaining the NP concentration at 80 wt%, the transfer fidelity obtained with each solvent is assessed by varying only the solvent to demonstrate how the interaction between the solvent and PDMS affects the transfer fidelity.

PDMS, which is composed of the recurring unit -Si(CH3)2-O-, is relatively nonpolar due to the well-balanced silicon-oxygen bonds, thus lacking distinct anodic or cathodic properties. MEK and acetone tend to be polar, with polarity indices of 4.7 and 5.1, respectively (Table S2). These solvents have moderate to relatively high polar contributions. In contrast, toluene and n-hexane are nonpolar with polarity indices of 2.4 and 0.0, respectively. Additionally, MEK, acetone, toluene, and n-hexane have solubility parameters of 9.3, 9.9, 8.9, and 7.3 cal^1/2^cm^−3/2^, respectively^51^. This parameter can be described as the sum of the dispersion, polar, and hydrogen bonding forces within the material. Solvents that have a solubility parameter similar to that of PDMS (7.3 cal^1/2^cm^−3/2^) generally cause PDMS to swell. MEK and acetone, which have low to moderate solubility, result in a low swelling effect. However, toluene and n-hexane, which have a high solubility for PDMS due to dispersion forces, result in a significant swelling ratio.

To intuitively demonstrate the swelling effect, real-time optical microscope (OM) is employed to observe the swelling of a pattern cross-section when the PDMS soft mold comes into contact with a solvent (Figs. S3, S4). When toluene and n-hexane, which have swelling ratios greater than that for MIBK, are dropped onto the soft mold and absorbed, the surface expands, and the desired pattern is distorted, while MEK and acetone do not transform the pattern. Therefore, when a nanocomposite film with a high swelling ratio is coated on the soft mold, the transfer fidelity significantly decreases.

**Fig. 5:**
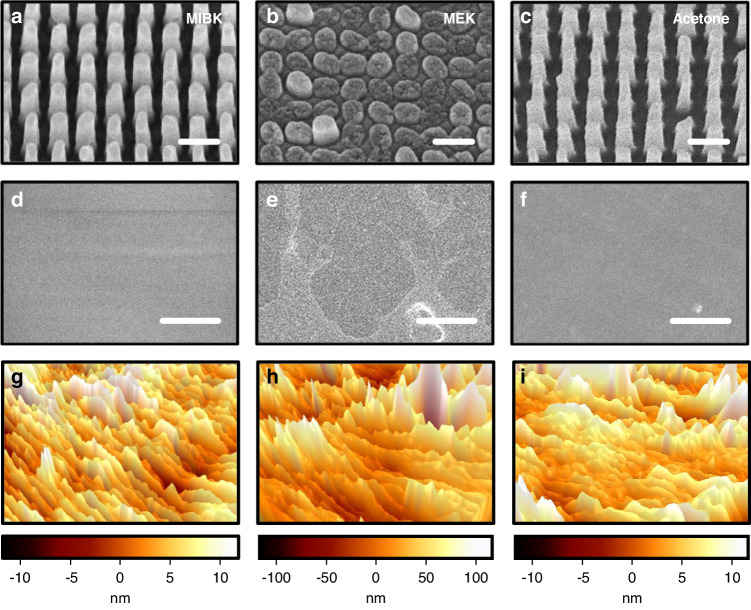
UV metaholograms replicated using the ZrO_2_ nanocomposites with different solvents of **a** MIBK, **b** MEK, and **c** acetone. (Scale bars: 300 nm.) SEM and atomic force microscope (AFM) images of soft molds after curing ZrO_2_ nanocomposites with solvents of **d**, **g** MIBK, **e**, **h** MEK, and **f**, **i** acetone. (Scale bars: 3 μm)

The pattern transfer fidelities for the various solvents are directly compared (Figs. 5, S5). When the MIBK-based nanocomposite is used, slight PDMS swelling occurs, but exceptional transfer fidelity can be achieved (Fig. 5a). However, application of the MEK-based nanocomposite under identical conditions yields a notably worse transfer fidelity despite it exhibiting a lower swelling ratio (Fig. 5b). The acetone-based nanocomposite yields high pattern definition, comparable to that with MIBK (Fig. 5c). The main factor contributing to the unexpected poor replication when using MEK is the interaction between MEK and PDMS, which leads to alterations in the surface segregation^52^, ultimately causing an increase in the surface roughness. To evaluate this phenomenon, ZrO_2_ nanocomposites based on MIBK, MEK, and acetone are applied and cured in the soft mold. The results show that MIBK exhibits a roughness distribution ranging from -10 to 10 nm (Fig. 5d, g), while that of MEK ranges from -100 to 100 nm (Fig. 5e, h), and that of acetone ranges from -10 to 10 nm (Fig. 5f, i). The measured surface roughness of the MIBK- and acetone-based nanocomposites is comparable to the diameter of the ZrO_2_ NPs ( ~ 15 nm), indicating a uniformly coated film. In contrast, the MEK-based nanocomposite results in a notably rough surface, causing a non-uniform coating that leads to irregularities in the transferred pattern. This non-uniform surface results in low transfer fidelity and diminishes the accuracy of the fine pattern details. Consequently, the MEK-based nanocomposite does not uniformly disperse across the soft mold, causing an uneven pressure distribution and inadequate formation of nanostructures during NIL. These findings indicate that in addition to causing swelling, the solvent also influences the surface roughness of the soft mold, which must also be considered. We highlight the crucial role of solvent selection in the NIL process for achieving high-fidelity metasurfaces.

**Fig. 6:**
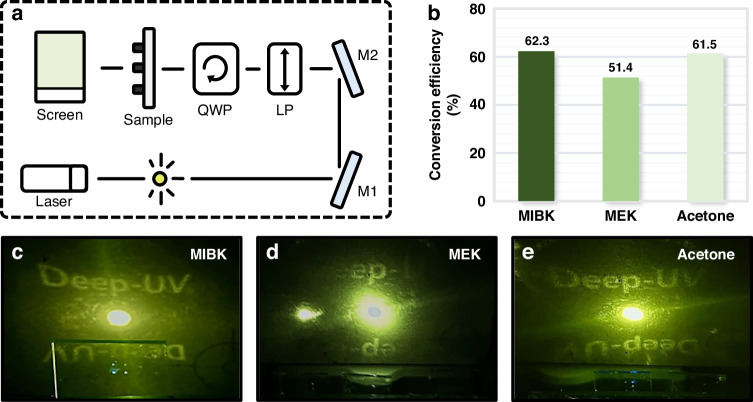
**a** Optical setup for UV metaholograms based on ZrO_2_ nanocomposites (Laser: 325 nm helium cadmium (HeCd) laser; M1, M2: mirrors; LP: linear polarizer; QWP: quarter-wave plate; Screen: UV detector card). **b** Measured conversion efficiencies corresponding to different ZrO_2_ nanocomposite solvents. Images measured at λ = 325 nm of UV metahologram obtained using **c** MIBK, **d** MEK, and **e** acetone as ZrO_2_ nanocomposite solvents

To quantify the transfer fidelity of nanocomposites with different solvents, UV metaholograms are evaluated based on the conversion efficiency, which is defined as the ratio between the powers of the incident light and converted light (Table S3). A helium cadmium (HeCd) laser (λ = 325 nm) is incident on the UV metaholograms. To achieve a circularly polarized beam, two UV wave plates, a linear polarizer, and a quarter-wave plate are employed (details in *Materials and Methods*, Fig. 6a). The UV metaholograms are visualized using a UV sensor card, and conversion efficiency is measured using a power meter. The MIBK- and acetone-based nanocomposites yield conversion efficiencies of 62.3% and 61.5%, respectively, whereas the MEK-based nanocomposite exhibits a lower conversion efficiency of 51.4% (Fig. 6b). The UV metaholograms replicated with the MIBK-based (Fig. 6c) and acetone-based (Fig. 6e) nanocomposites exhibit much clearer and more vibrant images than those replicated with the MEK-based nanocomposite (Fig. 6d). These results highlight that acetone demonstrates high transfer fidelity, leading to a performance comparable to that of MIBK.

## Discussion

In summary, we explored tailoring ZrO_2_ nanocomposites to manufacture high-fidelity UV metaholograms using NIL. By varying the ZrO_2_ NP concentration and investigating different solvents in the nanocomposite, the optimal conditions were identified. We determined that an 80 wt% ZrO_2_ NP concentration offered the most favorable conditions for producing metaholograms, yielding both high *n* and high transfer fidelity. An insufficient NP concentration led to reduced pattern definition and transfer fidelity due to inadequate mechanical support during NIL. Conversely, an excessive NP concentration caused particle agglomeration within the nanocomposite, leading to NP clusters larger than the cavity size in the soft mold. Furthermore, while maintaining an 80 wt% concentration, the interactions between different solvents and PDMS were also examined with regard to transfer fidelity. To intuitively demonstrate the swelling effect, we fabricated microscale soft molds and evaluated the degree of pattern distortion caused by the absorption of each solvent. Although both acetone- and MEK-based nanocomposites demonstrated a lower swelling ratio than MIBK, only acetone achieved successful pattern transfer with high fidelity. MEK unfavorably modified the PDMS surface properties, creating surface segregation and altering the solubility, resulting in increased surface roughness of the soft mold. This yielded an uneven nanocomposite coating, leading to non-uniform pressure on the substrate, which adversely affected the transfer fidelity and diminished the accuracy of the fine pattern details. Consequently, the MEK-based nanocomposite did not uniformly spread across the soft mold, significantly reducing the pattern definition and hindering the formation of meta-atoms. The conversion efficiency of metaholograms created using MIBK-, acetone-, and MEK-based nanocomposites was measured to quantify the transfer fidelity. The metaholograms replicated using MIBK- and acetone-based nanocomposites exhibited much clearer and more vibrant images than those replicated using MEK, with measured conversion efficiencies of 62.3%, 61.5%, and 51.4%. These results emphasize that acetone, in addition to MIBK, holds potential as a promising solvent for high-*n* nanocomposites to realize high-fidelity metasurfaces using NIL. These findings hold significant promise for optimizing the fabrication process and broadening the potential applications of nanocomposites in NIL. While the concept of ZrO_2_ nanocomposites has been previously introduced, there has been a lack of research explicitly detailing how factors such as the NP concentration and solvent in the nanocomposites precisely influence the outcomes of the NIL process. This study specifically addresses the influence of these factors, revealing potential advancements in NIL. Such UV metasurfaces composed of ZrO_2_ nanocomposite structures fabricated using a straightforward printing method offer additional benefits, such as a high throughput and a low fabrication cost. The integration of nanocomposite meta-atoms with conventional tuning techniques enables the development of multifunctional metaholograms, highlighting their potential for incorporation into holographic display technology and applications with augmented and virtual reality devices.

## Materials and methods

### Materials

ZrO_2_ nanocomposites were prepared from a solution with 30 wt% 10 nm ZrO_2_ NPs dispersed in MIBK, which were purchased from Ditto Technology (DT-ZROSOL-30MIBK (N10)). Dipentaerythritol penta-/hexa-acrylate and 1-hydroxycyclohexyl phenyl ketone were purchased from Sigma‒Aldrich and used as a monomer and a photoinitiator, respectively. MIBK, MEK, and acetone were purchased from Duksan General Science. h-PDMS, a vinylmethyl copolymer (VDT-731), a platinum catalyst (SIP6831.2), and a siloxane-based silane reducing agent (HMS-301) were purchased from Gelest. 2,4,6,8-Tetramethyl-2,4,6,8-tetravinylcyclotetrasiloxane and toluene, which were used as a modulator and a solvent, were purchased from Sigma‒Aldrich and Samchun Chemicals, respectively. PDMS, a silicone elastomer base, and a silicone elastomer curing agent were purchased from Dow Corning (SYLGARD™ 184 Silicone Elastomer Kit). The trichloro(1H,1H,2H,2H-perfluorooctyl)silane used for hydrophobic coating was purchased from Sigma‒Aldrich. Toluene and n-hexane were purchased from Samchun Chemicals.

### Fabrication of the master mold

The fabrication process began with a Si substrate as the master mold. Meta-atoms were then transferred onto a bilayer of two positive tone photoresists (495 PMMA A6, MicroChem & 950 PMMA A2, MicroChem) using the standard electron beam lithography (EBL) process with an ELIONIX ELS-7800 system operating at an acceleration voltage of 80 kV and a beam current of 100 pA. After exposure, the patterns were developed using a mixed solution of MIBK/IPA (1:3). An 80 nm chromium (Cr) layer was then deposited on the photoresist patterns through electron beam evaporation (KVT, KVE-ENS4004). The lifted-off Cr meta-atoms then served as an etching mask for the Si substrate. Transfer of Cr patterns onto the Si substrate was achieved using a dry etching process with a silicon/metal hybrid etcher (DMS). Finally, the remaining Cr etching mask was removed using a suitable Cr etchant (CR-7).

### Fabrication of the soft mold

A h-PDMS solution was prepared by mixing 3.4 g of the vinylmethyl copolymer, 18 μL of the platinum catalyst, 0.1 g of the modulator, 2 g of toluene, and 1 g of the siloxane-based silane reducing agent. The solution was spin-coated on the master mold at 2000 rpm for 60 s and then cured at 70 °C for 2 h. After baking, degassed PDMS solution, in which the silicone elastomer base and its curing agent were mixed at a weight ratio of 10:1, was poured on the h-PDMS layer and baked at 70 °C for 2 h. The cured soft mold was removed from the original master mold. To enable easy separation from the cured structure, the surface of the detached mold was coated with a hydrophobic self-assembled monolayer (SAM).

### Preparation of ZrO_2_ nanocomposites

In each chosen solvent, a monomer and a photoinitiator were dissolved at concentrations of 7 wt% and 3 wt%, respectively. DT-ZROSOL-30MIBK (N10) was used as the NP solution without any modifications to the solvent. The diluted or dispersed solutions were blended in the required proportions at the desired ratio.

### Nanoimprint lithography process

To improve the adhesion of the substrate, a PMMA solution was carefully applied to the cleaned and oxygen plasma-treated surface, resulting in a hydrophilic modification. Next, the ZrO_2_ nanocomposite, which consisted of a monomer, an initiator, and NPs, was gently drop-coated onto the soft mold. The coated mold was left at room temperature for approximately 5 min, enabling the nanocomposite solvent to spontaneously evaporate. Finally, the coated mold was cured under 5 bar of pressure and 15 min of UV irradiation.

### Swelling experiments

The initial weight of the PDMS elastomers was measured prior to immersion in solvents. Approximately 30 mL of each solvent was placed in separate beakers. The PDMS elastomers were then immersed in the individual solvents for 15 min. The weight of the swollen PDMS elastomers was subsequently measured. This process involved recording the weight of each PDMS elastomer both before and after immersion. The swelling ratio, defined as the ratio between the weight of the swollen PDMS and its initial dry weight, was calculated. These PDMS swelling experiments were repeated five times for each solvent.

### Optical measurement

The beam of a 325 nm laser (helium cadmium laser, Kimmon Koha Co., Ltd.) was passed through a linear polarizer (linear polarizer, Thorlabs) and a quarter-wave plate (quarter-wave plate, Thorlabs) to form circularly polarized light. A 500 µm diameter pinhole [P500HD – Ø1/2 in. (12.7 mm) mounted pinhole, Thorlabs] was used to block unnecessary light. A photodiode power sensor (S120C, Thorlabs) and a compact power and energy meter console (PM100D, Thorlabs) were used to measure the intensity of the light. Holographic images were extracted through a UV sensor card [laser viewing card (VRC1), Thorlabs].

### Supplementary information

Supplementary information
