# Supplementary material for: Tailoring high-refractive-index nanocomposites for manufacturing of ultraviolet metasurfaces
Source: Microsyst Nanoeng. 2024 Apr 22;10:53. doi: 10.1038/s41378-024-00681-w (PMC11035676; doi:10.1038/s41378-024-00681-w)
Supplement: Supplementary file 1 — Supplementary information [file 41378_2024_681_MOESM1_ESM.pdf]

Supplementary Information

Tailoring high-refractive-index nanocomposites for the manufacturing of ultraviolet metasurfaces

Hyunjung Kang[1], Dongkyo Oh[1], Nara Jeon[1], Joohoon Kim[1], Hongyoon Kim[1], Trevon Badloe[3], and Junsuk Rho[1,2,4,5,6]*

[1]Department of Mechanical Engineering, Pohang University of Science and Technology (POSTECH), Pohang 37673, Republic of Korea

[2]Department of Chemical Engineering, Pohang University of Science and Technology (POSTECH), Pohang, 37673, Republic of Korea

[3]Graduate School of Artificial Intelligence, Pohang University of Science and Technology (POSTECH), Pohang, 37673, Republic of Korea

[4]Department of Electrical Engineering, Pohang University of Science and Technology (POSTECH), Pohang, 37673, Republic of Korea

[5]POSCO-POSTECH-RIST Convergence Research Center for Flat Optics and Metaphotonics, Pohang 37673, Republic of Korea

[6]National Institute of Nanomaterials Technology (NINT), Pohang 37673, Republic of Korea

*Corresponding author. E-mail: jsrho@postech.ac.kr

Supplementary Note 1. Jones matrix analysis

To achieve the Pancharatnam-Berry (PB) phase, anisotropic meta-atoms are used. These meta-atoms are represented using a Jones matrix **J**, which can be expressed as follows:

$$\mathbf{J} = \begin{bmatrix} t_{xx} & 0 \\ 0 & t_{yy} \end{bmatrix},$$

where t_{xx} and t_{yy} represent its complex transmission coefficients for longer and shorter axes of the rectangular nanorod, respectively. The Jones matrix **T** for a rectangular nanorod, rotated at an angle θ, can be determined using a rotation matrix **R**(θ) as

$$\mathbf{T} = \mathbf{R}(-\theta)\,\mathbf{J}\,\mathbf{R}(\theta).$$

This matrix can be expressed as

$$\boldsymbol{E_t} = \frac{t_{xx}+t_{yy}}{2}\begin{bmatrix} 1 & 0 \\ 0 & \pm i \end{bmatrix} + \frac{t_{xx}-t_{yy}}{2} e^{i2\theta} \begin{bmatrix} 1 & 0 \\ 0 & \mp i \end{bmatrix}.$$

Supplementary Note 2. Measured ellipsometry results

The refractive indices of the ZrO_2 nanocomposite film according to NP concentrations were measured using ellipsometry. The measured amplitude ratio (Ψ) and phase difference (Δ) of ZrO_2 nanocomposite films were fitted using Tauc-Lorentz model.

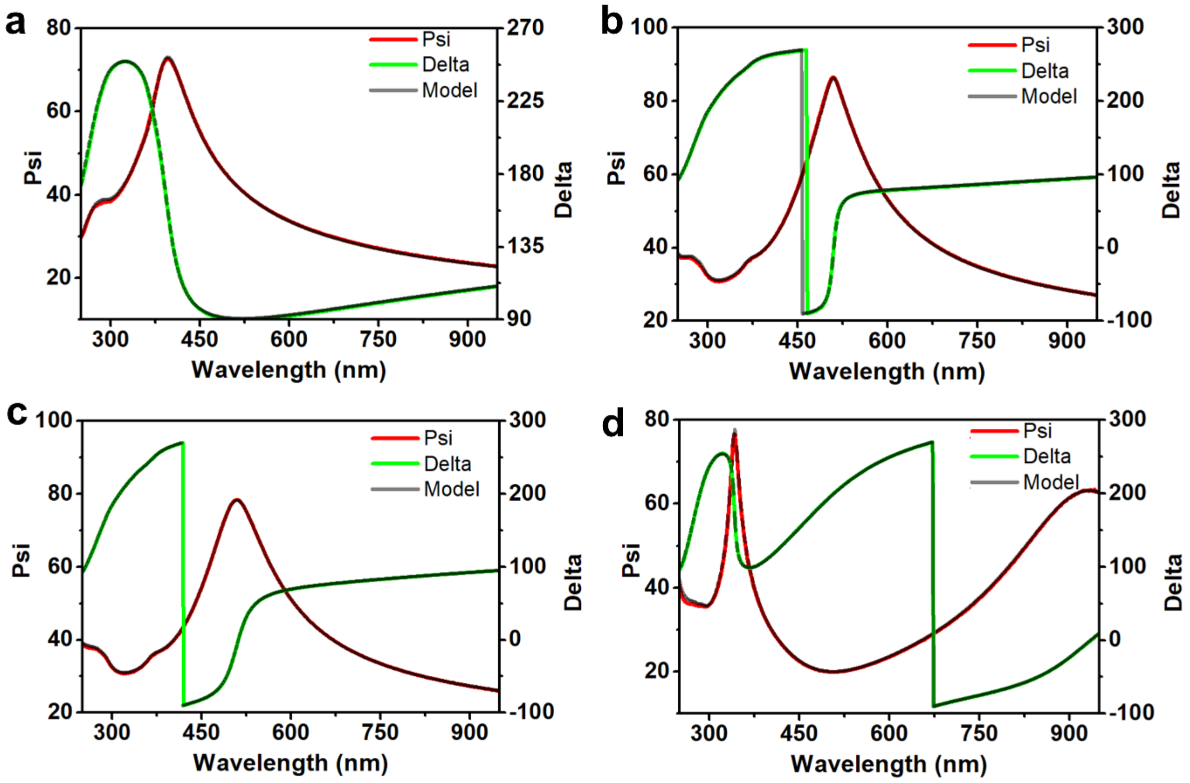

Fig. S1 Measured amplitude ratio (Ψ) and phase difference (Δ) of the zirconium dioxide (ZrO_2) nanocomposite film according to NP concentrations: **(a)** 20 wt%; **(b)** 50 wt%; **(c)** 80 wt%; **(d)** 90 wt%.

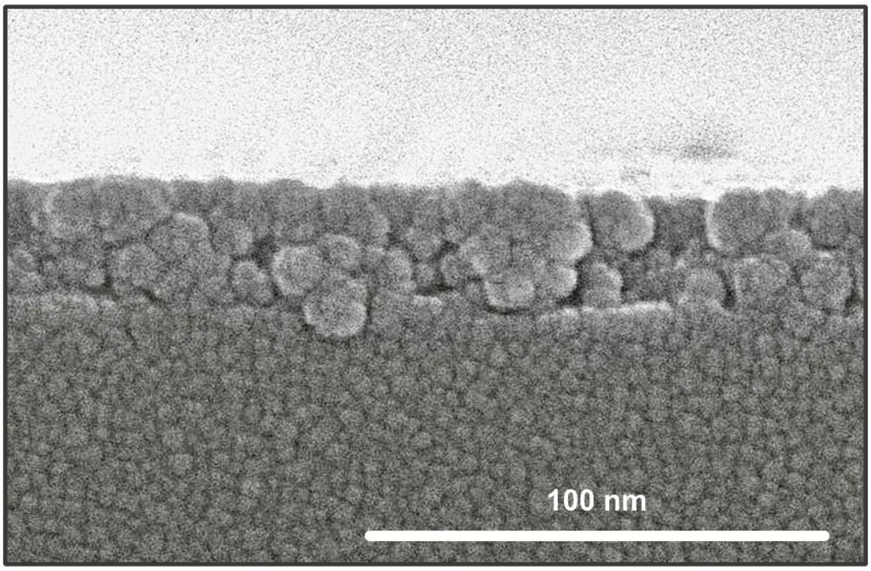

Fig. S2 A scanning electron microscope (SEM) image of a spin-coated 80wt% ZrO_2 nanocomposite film on a glass substrate.

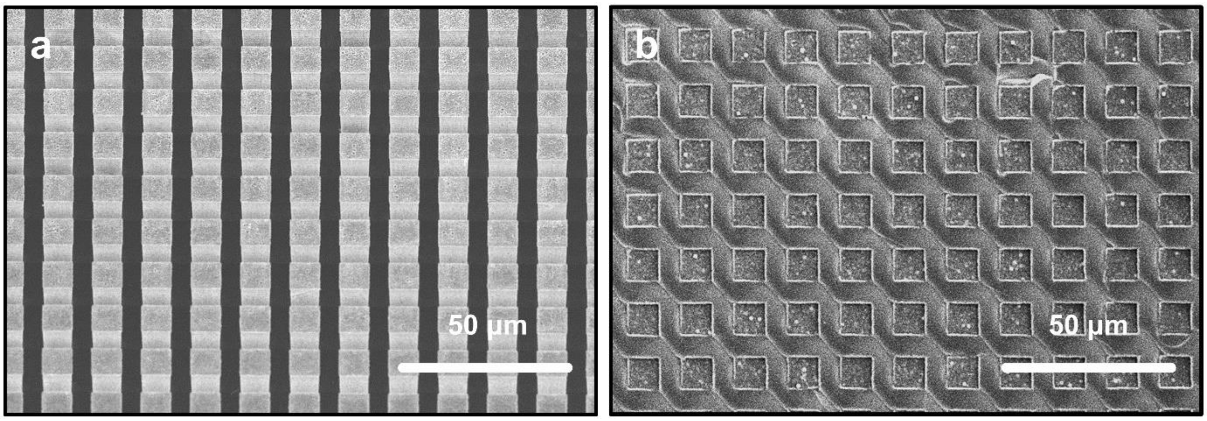

Fig. S3 SEM images of microscale **(a)** master mold and **(b)** soft mold.

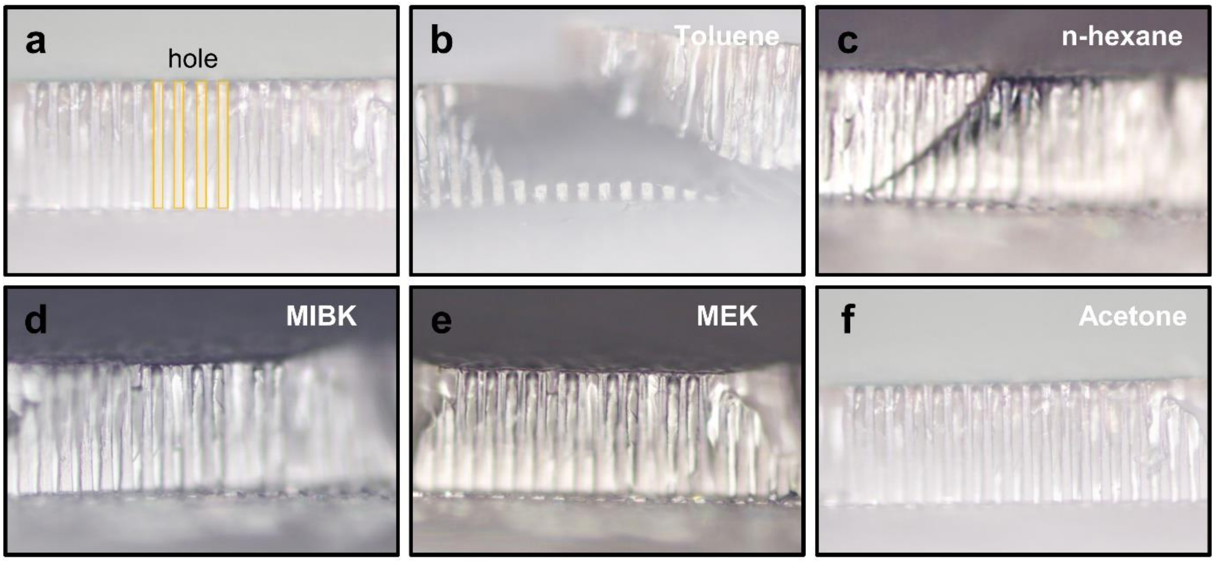

Fig. S4 Optical microscope (OM) images of the cross-section microscale soft mold **(a)** before coming into contact with the solvents, and after coming into contact with **(b)** toluene, **(c)** n-hexane, **(d)** methyl isobutyl ketone (MIBK), **(e)** methyl ethyl ketone (MEK), and **(f)** acetone.

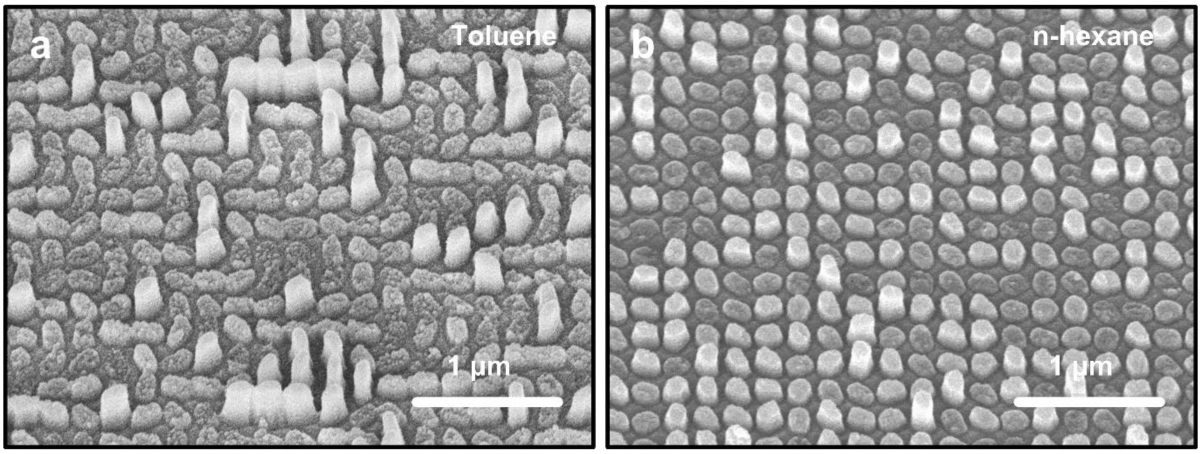

Fig. S5 Replicated ultraviolet (UV) metaholograms using the ZrO_2 nanocomposite with solvents of **(a)** toluene and **(b)** n-hexane.

Supplementary Note 3. PDMS-solvent interaction

Polydimethylsiloxane (PDMS), a component of the soft mold used in nanoimprint lithography, is a non-polar polymer composed mainly of silicon and oxygen atoms. Due to its non-polar nature and the relatively high force of polymerization during its formation, the PDMS surface exhibits low interaction with polar substances, such as water, while displaying higher interaction with gaseous substances, organic solvents, or other non-polar substances. As a result, the swelling ratio of PDMS decreases as the polarity index of the solvent increases.

Table S1. Calculation of the swelling ratio for different solvents.

Solvent type	Dry PDMS weight (g)	Swollen PDMS weight (g)	Swelling ratio
MIBK	1.503	1.732	1.152
Acetone	1.508	1.618	1.073
MEK	1.342	1.489	1.11
Toluene	1.344	1.843	1.371
n-hexane	1.502	2.073	1.38

Table S2. Polarity index of solvents

**Solvent**	Acetone	MIBK	MEK	Toluene	n-hexane
**Polarity index**	5.1	4.2	4.7	2.4	0.0

Table S3. Calculation of the measured conversion efficiency

**Solvent type**	**Reference (μW)**	**Converted beam (μW)**	**Conversion efficiency (%)**
MIBK	3.2	2.14	66.9
Acetone	3.9	2.4	61.5
MEK	3.6	1.85	51.4
